# Enhanced Motor Imagery-Based BCI Performance via Tactile Stimulation on Unilateral Hand

**DOI:** 10.3389/fnhum.2017.00585

**Published:** 2017-12-01

**Authors:** Xiaokang Shu, Lin Yao, Xinjun Sheng, Dingguo Zhang, Xiangyang Zhu

**Affiliations:** ^1^State Key Laboratory of Mechanical System and Vibration, Shanghai Jiao Tong University, Shanghai, China; ^2^Department of Systems Design Engineering, Faculty of Engineering, University of Waterloo, Waterloo, ON, Canada

**Keywords:** motor imagery (MI), brain-computer interface (BCI), tactile stimulation, event-related de/synchronization (ERD/ERS), event-related spectral perturbation (ERSP)

## Abstract

Brain-computer interface (BCI) has attracted great interests for its effectiveness in assisting disabled people. However, due to the poor BCI performance, this technique is still far from daily-life applications. One of critical issues confronting BCI research is how to enhance BCI performance. This study aimed at improving the motor imagery (MI) based BCI accuracy by integrating MI tasks with unilateral tactile stimulation (Uni-TS). The effects were tested on both healthy subjects and stroke patients in a controlled study. Twenty-two healthy subjects and four stroke patients were recruited and randomly divided into a control-group and an enhanced-group. In the control-group, subjects performed two blocks of conventional MI tasks (left hand vs. right hand), with 80 trials in each block. In the enhanced-group, subjects also performed two blocks of MI tasks, but constant tactile stimulation was applied on the non-dominant/paretic hand during MI tasks in the second block. We found the Uni-TS significantly enhanced the contralateral cortical activations during MI of the stimulated hand, whereas it had no influence on activation patterns during MI of the non-stimulated hand. The two-class BCI decoding accuracy was significantly increased from 72.5% (MI without Uni-TS) to 84.7% (MI with Uni-TS) in the enhanced-group (*p* < 0.001, paired *t*-test). Moreover, stroke patients in the enhanced-group achieved an accuracy >80% during MI with Uni-TS. This novel approach complements the conventional methods for BCI enhancement without increasing source information or complexity of signal processing. This enhancement via Uni-TS may facilitate clinical applications of MI-BCI.

## 1. Introduction

Brain-computer interface (BCI) provides a non-muscular communication and control channel for a human brain to directly interact with the external world (Wolpaw et al., 2002). BCI users can initiate brain-control by simply performing left- or right-hand motor imagery (MI). The kinesthetic imagination of hand movement generates event-related (de)synchronizations (ERD/ERS) in subject's sensorimotor cortex (Pfurtscheller and Neuper, 1997; Pfurtscheller and Lopes da Silva, 1999; Pfurtscheller, 2000). ERD (ERS) activities are defined as the percentage of power decrease (increase) in a specific frequency band (e.g., alpha band [8 13] Hz, or beta band [14 30] Hz) in relation to a reference interval (Graimann et al., 2002). Typically, MI-induced ERD activities are lateralized to the contralateral hemisphere, i.e., right-hand MI induces ERD in the left sensorimotor cortex, whereas ERD appears in the right sensorimotor cortex during left-hand MI (Pfurtscheller et al., 1997). Accordingly, these lateralized cortical activities constitute the neurophysiological basis of motor imagery-based BCI (MI-BCI) (Pfurtscheller and Neuper, 2001; Blankertz et al., 2008a, 2010).

However, there exist spatially asymmetrical cortical activations between left- and right-hand MI in some cases. In stroke patients, paretic hand MI ability greatly decreases due to the motor deficit (de Vries et al., 2011). It leads to abnormal cortical activation patterns. During paretic hand MI, stronger ERD activities appear in the ipsilateral (intact) hemisphere rather than in the contralateral (lesioned) hemisphere (Park et al., 2016). A near-infrared spectroscopy (NIRS) study found that movement intention of the paretic hand activated bilateral sensorimotor cortices in stroke patients, and the lateralization of cortical activations was significantly decreased (Takeda et al., 2007). Even in healthy subjects, handedness also results in cortical difference through gating effects (Tecchio et al., 2006). It has been demonstrated that MI abilities are unbalanced between dominant and non-dominant hands (Maruff et al., 1999). The dominant hand usually shows better performance in MI tasks (e.g., faster, or more accurate) (Guillot et al., 2010; Paizis et al., 2014). This behavioral difference produces asymmetrical cortical activations between dominant and non-dominant hand MI. Magnetoencephalography (MEG) study indicated that non-dominant hand MI induced stronger beta ERD in the ipsilateral sensorimotor cortex than in the contralateral sensorimotor cortex (Boe et al., 2014). Previous research using transcranial magnetic stimulation (TMS) also exhibited significantly higher motor-evoked potential (MEP) amplitudes during the dominant hand MI compared to the non-dominant hand MI (Stinear et al., 2006; Gandrey et al., 2013). Therefore, it is speculated that the asymmetrical cortical activations between left- and right-hand MI may reduce the ERD lateralization, and consequently affect the two-class BCI decoding accuracy.

Some evidence indicates that integrating MI with tactile stimulation improves MI abilities. A study on tennis players showed that holding a tennis racket as sensory feedback for MI was able to significantly decrease the imagining time compared with the MI when holding nothing (Mizuguchi et al., 2015). Furthermore, physiological studies found that tactile stimulation applied on the imagined hand could enhance the contralateral cortical activations (Mizuguchi et al., 2009, 2013), but no cortical effect was found when the stimulation was applied on the opposite hand (Mizuguchi et al., 2012). This evidence suggests that unilateral tactile stimulation (Uni-TS) on non-dominant/paretic hand has the potential to increase MI-induced ERD lateralization, whereby the Uni-TS provides a way to improve MI-BCI decoding accuracy.

In this work, we explored the influence of Uni-TS on MI-induced cortical oscillations and on two-class MI-BCI performance (left hand vs. right hand). Both healthy subjects and stroke patients were recruited to evaluate the Uni-TS aided BCI system. More specifically, constant vibrotactile stimulations were applied on the paretic hand of stroke patients or the non-dominant hand of healthy subjects during both left- and right-hand MI. Cortical activations during MI tasks with and without stimulation were qualitatively analyzed, and two-class BCI performances in different conditions were evaluated with on-line and off-line pattern classification.

## 2. Materials and methods

### 2.1. Subjects

Twenty-two healthy subjects (all right-handed, 6 females, average of 22 ± 1.5 years) and four stroke patients (2 females) were recruited for this study. Both healthy and stroke participants were BCI-naive subjects. The hand dominances of healthy subjects were determined by the Edinburgh Handedness inventory (Oldfield, 1971). Characteristics of recruited stroke patients are listed in Table 1. Subjects were randomly divided into two groups, the control-group and enhanced-group. Healthy subjects were labeled as HC1 to HC11 in the control-group, and HE1 to HE11 in the enhanced-group. Stroke patients were labeled as SC1 and SC2 in the control-group, and SE1 and SE2 in the enhanced-group. They were informed about the experimental protocol and required to sign informed consent forms before participation. The study was approved by the Ethical Committee of Shanghai Jiao Tong University, Shanghai, China.

**Table 1:** Characteristics of stroke patients.

**Index**	**Affected side**	**Post-stroke time (month)**	**Injury type**	**Lesion site**	**MMSE**	**up-FMA**
SC1	L	12	Ischemia	Cortical	30	19
SC2	R	4	Hemorrhage	Cortical	29	28
SE1	L	13	Hemorrhage	Cortical	28	32
SE2	R	3	Ischemia	Cortical	30	7

*MMSE, Mini-Mental State Examination*.

*up-FMA, Fugl-Meyer Assessment of upper limb*.

### 2.2. EEG recording and tactile stimulation device

EEG signal was recorded using a 64-channel Quik-Cap (Compumedics Neuroscan, Charlotte, NC, USA) and amplified using a SynAmps2 system (Compumedics Neuroscan, Charlotte, NC, USA). The electrodes were distributed according to the extended 10–20 system. The reference electrode was placed on the vertex, and the ground electrode was on the forehead. Impedances for all electrodes were maintained at <5 kΩ throughout the experiment. Raw signals were sampled at 250 Hz with an analog bandpass filter between 0.5 and 70 Hz. A 50 Hz notch filter was used to diminish power line interference.

**Figure 1:**
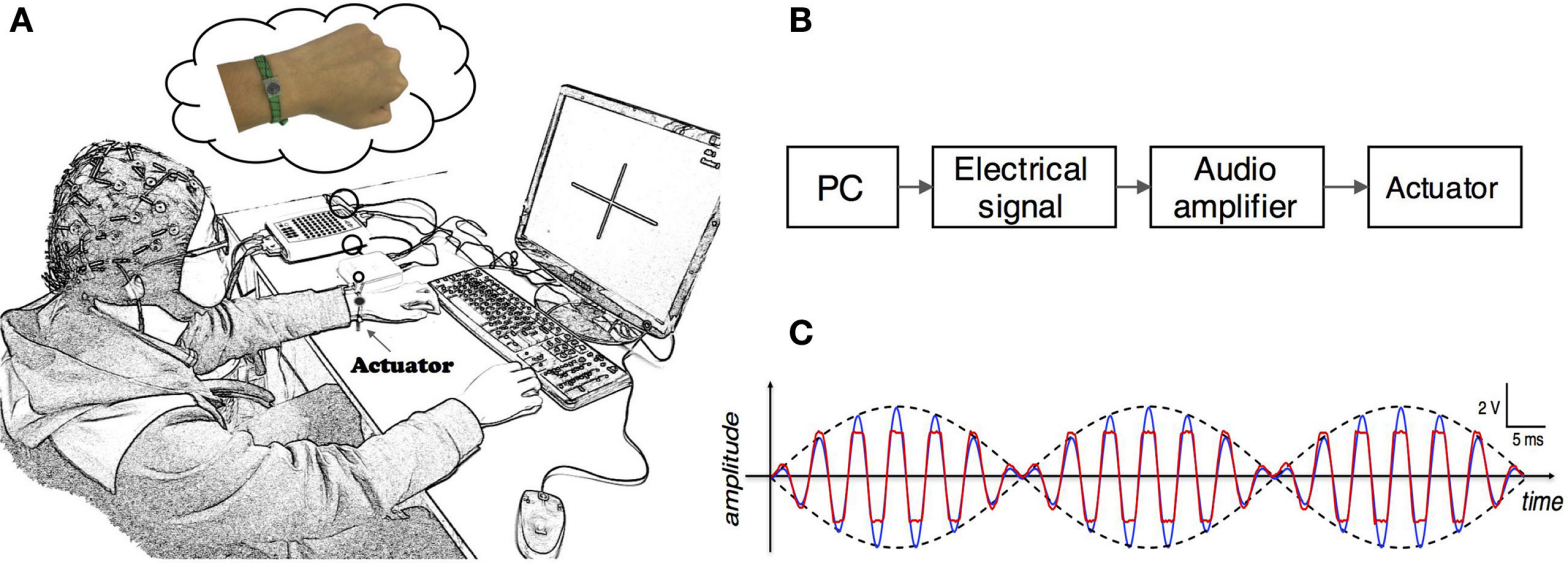
**(A)** Experimental setup. Subjects were seated in a comfortable chair with arms resting on the desk in the front. An actuator was attached to the wrist of non-dominant/paretic hand for tactile stimulation. The subjects were required to focus on the screen and avoid any muscular movements during mental tasks. **(B)** Electrical signals were produced via computer soundcard and amplified with an audio amplifier to drive the actuator. **(C)** Electrical signal of 27 Hz sinusoidal frequency modulated with 175 Hz sinusoidal carrier frequency. The blue and red curves are intended and measured signals, separately.

During the experiment, subjects were seated in a comfortable chair with arms resting on the desk in front of them as shown in Figure 1A. A linear resonant actuator (10 mm, C10-100, Precision Microdrives Ltd. Typical Normalized Amplitude 1.4G) was used for tactile stimulation. The actuator was positioned (close to distal radius and ulna) on the non-dominant wrist of healthy subjects or paretic wrist of stroke patients using a self-made wrist band. Electrical signal was produced via computer soundcard (Realtek High Definition Audio, REALTEK, Taiwan) and amplified with an audio amplifier (Serenade DSD TempoTec Corp, Beijing, China) to drive the actuator as the procedure shown in Figure 1B. The actuator produced a 27 Hz sine wave and it was modulated with a 175 Hz sine carrier wave as shown in Figure 1C. The tactile stimulation activated the Pacinian and Meissner corpuscles, which were sensitive to stimulations at frequencies above 100 Hz and 20–50 Hz, respectively (Kandel et al., 2000). The maximum amplitude of the stimulation was 11.3 μm. For each subject, the stimulation amplitude was optimized following three steps: (1) the experimenter slightly adjusted the stimulation amplitude and inquired the subjects whether they can obviously perceive the stimulation, (2) subjects were required to check whether they can freely perform MI tasks of non-stimulated hand, and (3) a 5-min stimulation with the selected amplitude was tested on subjects before experiment to ensure the stimulation produces no significant muscle fatigue. Experiments started only if the subjects reported “yes” to the first two questions and confirmed that no significant fatigue was produced by the stimulation.

### 2.3. Experimental paradigm

**Figure 2:**
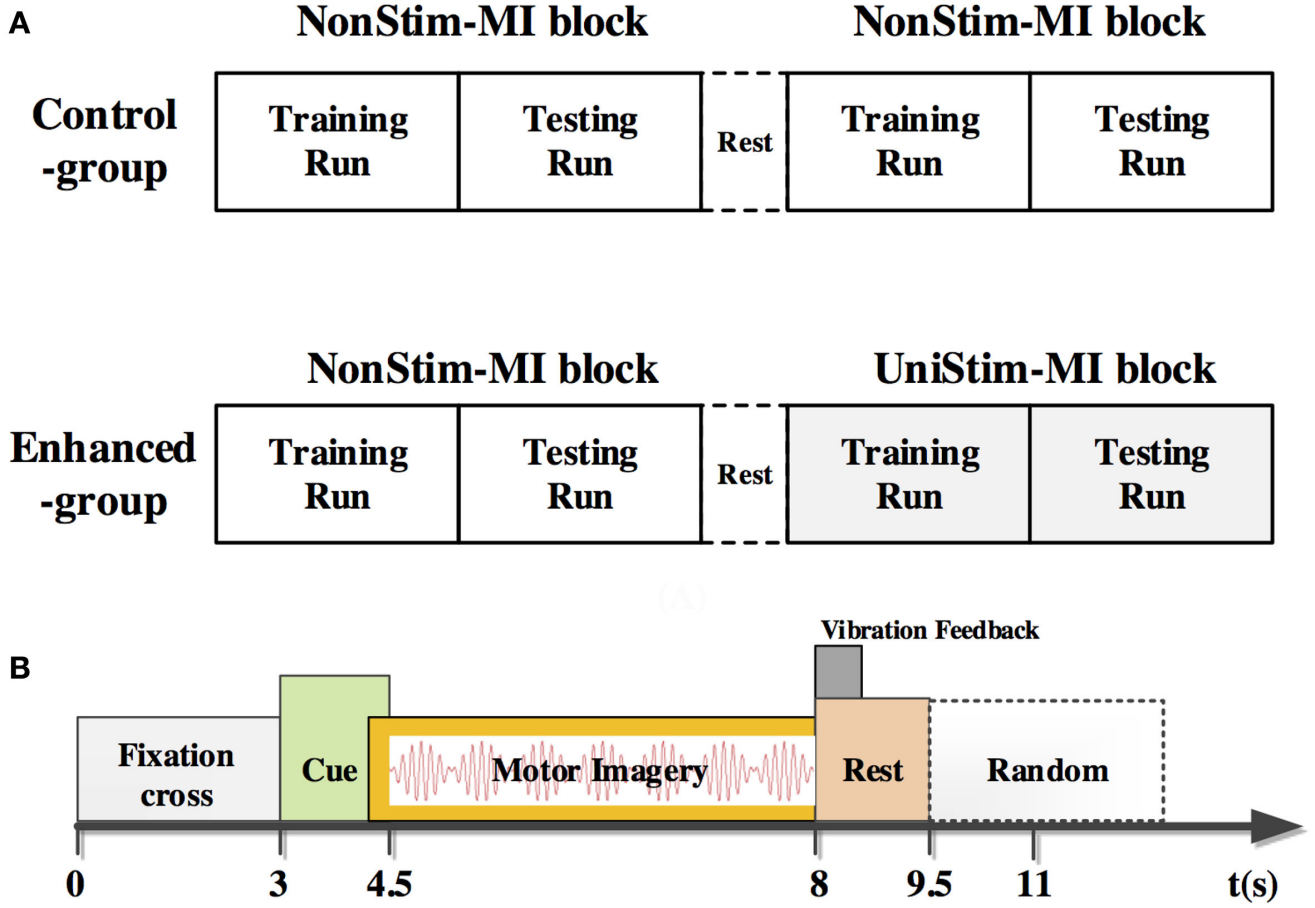
**(A)** Experimental procedure of the control-group and enhanced-group. Each group consisted of two blocks of MI. In the second block of the enhanced-group, there was a constant tactile stimulation applied on subject's non-dominant/paretic wrist, whereas no stimulation was applied in other MI blocks. The block with stimulation was named as UniStim-MI block, and the other blocks were named as NonStim-MI block. A short break was taken between two blocks. **(B)** Time structure of one single trial. A constant tactile stimulation was applied on non-dominant/paretic hand from 4.5 to 8 s in UniStim-MI block. Each run contained 20 trials of left-hand MI and 20 trials of right-hand MI.

Subjects completed mental tasks following the experimental protocol as shown in Figure 2A. There were two different experimental conditions. Tasks in the first condition were conventional MI of left and right hand without any stimulation, namely the non-stimulation MI (NonStim-MI) block. In the second condition there was a constant stimulation applied on the subject's non-dominant/paretic wrist during MI tasks, termed as the unilateral-stimulation MI (UniStim-MI) block. During MI tasks, subjects were instructed to mentally simulate the movement of wrist extension and flexion.

In the enhanced-group, one NonStim-MI block and one UniStim-MI block were performed. In each block, there were 40 trials in the first run, and followed by 40 trials in the second run during which on-line performance was tested by single-trial classification. During on-line testing, if the mental task was correctly classified, a word “right *N*” would appear at the upper-right corner of the screen immediately after each trial as feedback. Number “*N*” represented the amount of correctly classified trials. The time structure of a single trial is shown in Figure 2B. At the beginning of each trial, there was a white cross presented in the center of the screen to remind the subject to be prepared and keep still. At 3 s, a red cue appeared at left or right side of the cross, indicating the subject to perform a left- or right-hand MI. This visual cue disappeared after 1.5 s, but the mental task was required to continue until the white cross disappeared at 8 s. Then a short rest of 3.5–4.5 s was followed before the next trial. In the control-group, subjects were required to perform two blocks of NonStim-MI tasks. The time structure of each trial was the same with that of the enhanced-group, and the on-line test was performed in the second run of each block. The data from this group was utilized for inter- and intra- group comparisons to exclude the training effects on BCI performance.

### 2.4. Algorithms and analysis methods

Event-related spectral perturbation (ERSP) and ERD/ERS are commonly used to evaluate the spectral power changes originated from MI tasks (Makeig et al., 2004). The ERSP and ERD/ERS values were calculated within the alpha-beta frequency band of [8 30] Hz. The reference interval was −800 to −100 ms prior to the appearance of cues. ERD/ERS values at the critical channels of C3 and C4 were averaged among 1–4 s post-task cues. ERSP values were presented from both spatial and time-frequency domains. Time-frequency analysis of each trial was undertaken at channel C3 and C4 using EEGLAB toolbox (Delorme et al., 2011). It was calculated every 200 ms with a Hanning tapper, convoluted with a modified sinusoid basis in which the number of cycles linearly changed with frequency to achieve proper time and frequency resolution. Meanwhile, non-significant ERSP values were wiped out under bootstrap significance level of *p* = 0.01. Muscular artifacts were detected using *ft*_*artifact*_*zvalue* function in FieldTrip toolbox (Oostenveld et al., 2011). In this algorithm, *z*-values of each sampling point were calculated by subtracting the mean and dividing by the standard deviation. A threshold of *z*-value = 40 was used for automatically artifact rejection in which an average of 5.9 ± 4.4 (MEAN ± SD) trials were rejected for all participants.

EEG signals at 1–4 s post-task cues were extracted for pattern classification. The raw signals were bandpass filtered using a 4th-order Butterworth filter at 8–30 Hz. Then, common spatial pattern (CSP) algorithm was adopted for spatial filtering (Blankertz et al., 2008b), and linear discriminative analysis (LDA) (Pfurtscheller et al., 1998) was used as a classifier for both on-line and off-line decoding. During on-line classification, CSP and LDA parameters were calibrated at every trial, i.e., classification of the current trial was based on the parameters from all the previous trials. A 10 × 10-fold cross-validation was adopted in the off-line analysis. The 80 trials of MI were randomly separated into 10 portions with each one consisting of 8 trials. Nine portions of data were used for training the LDA classifier, while the left one portion was used for testing. This step was repeated 10 times to generate 100 results for statistical analysis.

The *R*^2^ index (squared Pearson-correlation coefficient between EEG features and class labels) (Schalk et al., 2004) was used to evaluate the contribution of different EEG channels for BCI classification. It was calculated based on the spatial-spectral-temporal structures of EEG signals from different mental tasks. With topographic *R*^2^ value distributions, we can have better understanding of the brain activation and its influence on pattern classification. Here, the *R*^2^-values were averaged along the timing interval of 1–4 s and frequency band of 8–30 Hz.

## 3. Results

### 3.1. Effects of Uni-TS on cortical oscillations

**Figure 3:**
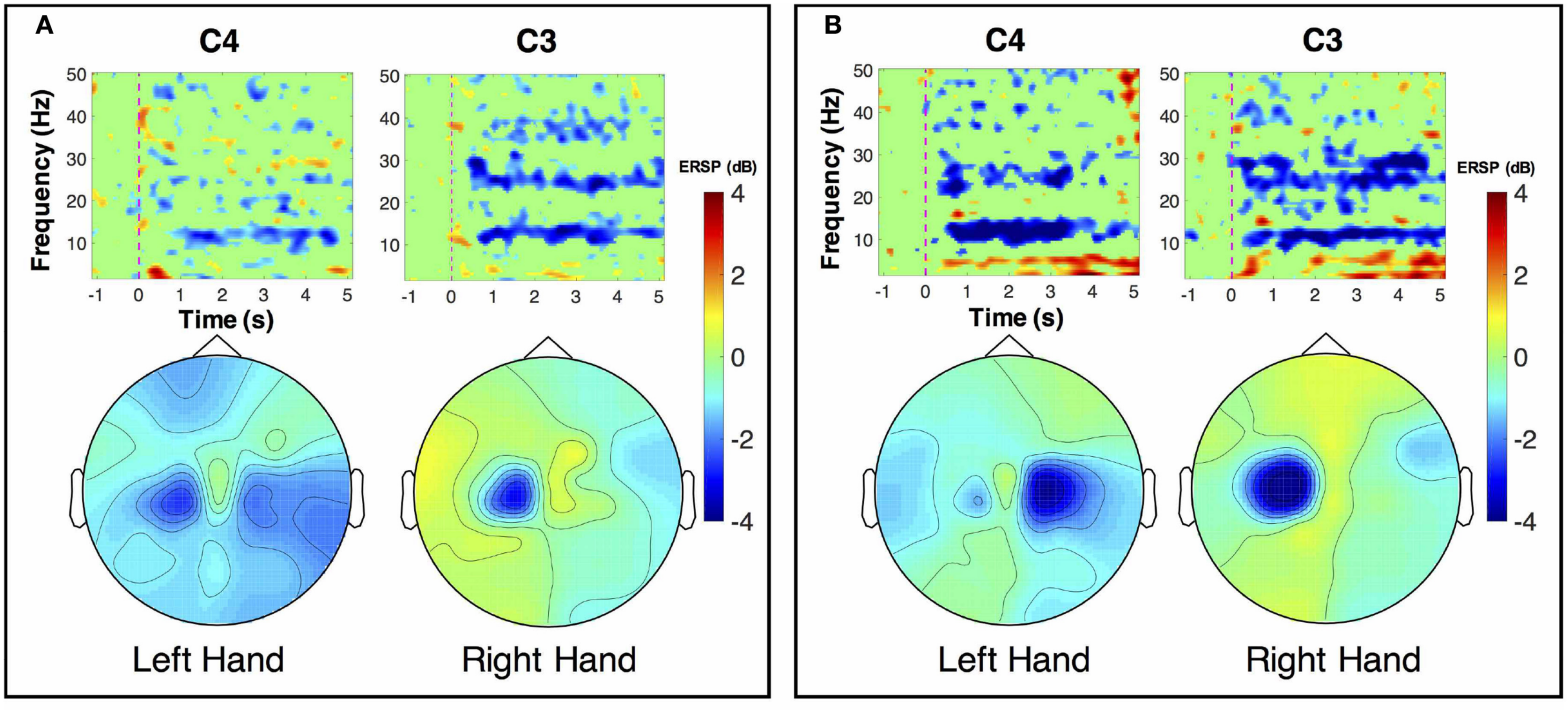
MI-induced cortical activations in time-frequency (upper row) and spatial (bottom row) domains for a healthy subject HE3. The topographic maps were averaged within the frequency band of [8 13] Hz, and timing interval of [1 4] s post-task cues. **(A)** Cortical activations during NonStim-MI. Ipsilateral cortical areas were activated during the left hand MI, and activation patterns were similar between the left- and right-hand MI. **(B)** Cortical activations during UniStim-MI. Contralateral motor cortex was obviously activated during the left-hand MI when Uni-TS was applied, but there was no effect on activation patterns during the right-hand MI. In the time-frequency maps, the vertical dashed red line indicates the appearance of task cues. Note that the blue color represents a power decrease and the red color represents a power increase.

**Figure 4:**
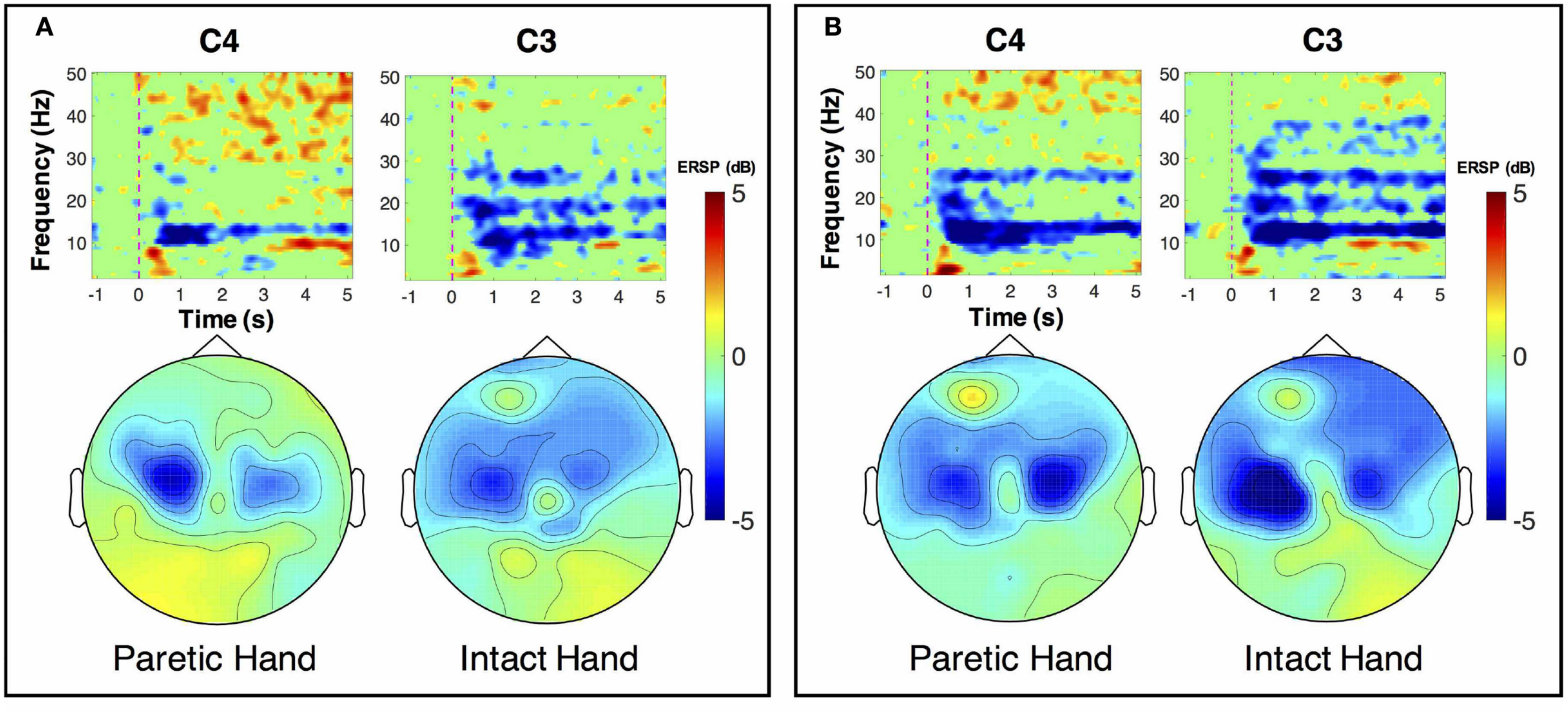
MI-induced cortical activations in time-frequency (upper row) and spatial (bottom row) domains for a stroke patient SE1, who was with paralysis on the left hand. The topographic maps were averaged within the frequency band of [8 13] Hz, and timing interval of [1 4] s post-task cues. **(A)** Cortical activations during NonStim-MI. Obvious activations were observed during both paretic and intact hand MI, but the activation patterns were similar between different MI tasks. **(B)** Cortical activations during UniStim-MI. When the Uni-TS was applied, cortical activations were enhanced in alpha frequency band during both paretic and intact hand MI. Moreover, the activations during paretic hand MI were lateralized to the contralateral side. In the time-frequency maps, the vertical dashed red line indicates the appearance of task cues. Note that the blue color represents a power decrease and the red color represents a power increase.

ERSP distributions across time-frequency and spatial domains from two representative subjects (HE3 and SE1) are shown in Figures 3, 4. Time-frequency analyses were performed at channel C3 in the left hemisphere (for right-hand MI) and channel C4 in the right hemisphere (for left-hand MI). For healthy subject HE3, the sensorimotor cortices (covered by C3 and C4 channels) were activated in alpha and beta frequency bands during NonStim-MI tasks as shown in Figure 3A. Meanwhile, the topographic maps showed the contralateral sensorimotor cortex was obviously activated during the right-hand MI, whereas the ipsilateral sensorimotor cortex exhibited stronger activations during the left-hand MI. When Uni-TS was applied on the left hand, the activations in alpha and beta frequency bands were enhanced at both channel C3 and C4 as shown in Figure 3B. Moreover, the topographic map corresponding to the left-hand MI indicated that cortical activations were lateralized to the contralateral side when the Uni-TS was applied. Figure 4 displays the results of stroke patient SE1. Obvious activations were observed during the NonStim-MI tasks of both hands, but the activations were not lateralized to the contralateral side during the paretic hand MI, as shown in Figure 4A. However, when the Uni-TS was applied, contralateral cortical activations were enhanced during both paretic and intact hand MI, as shown in Figure 4B.

**Figure 5:**
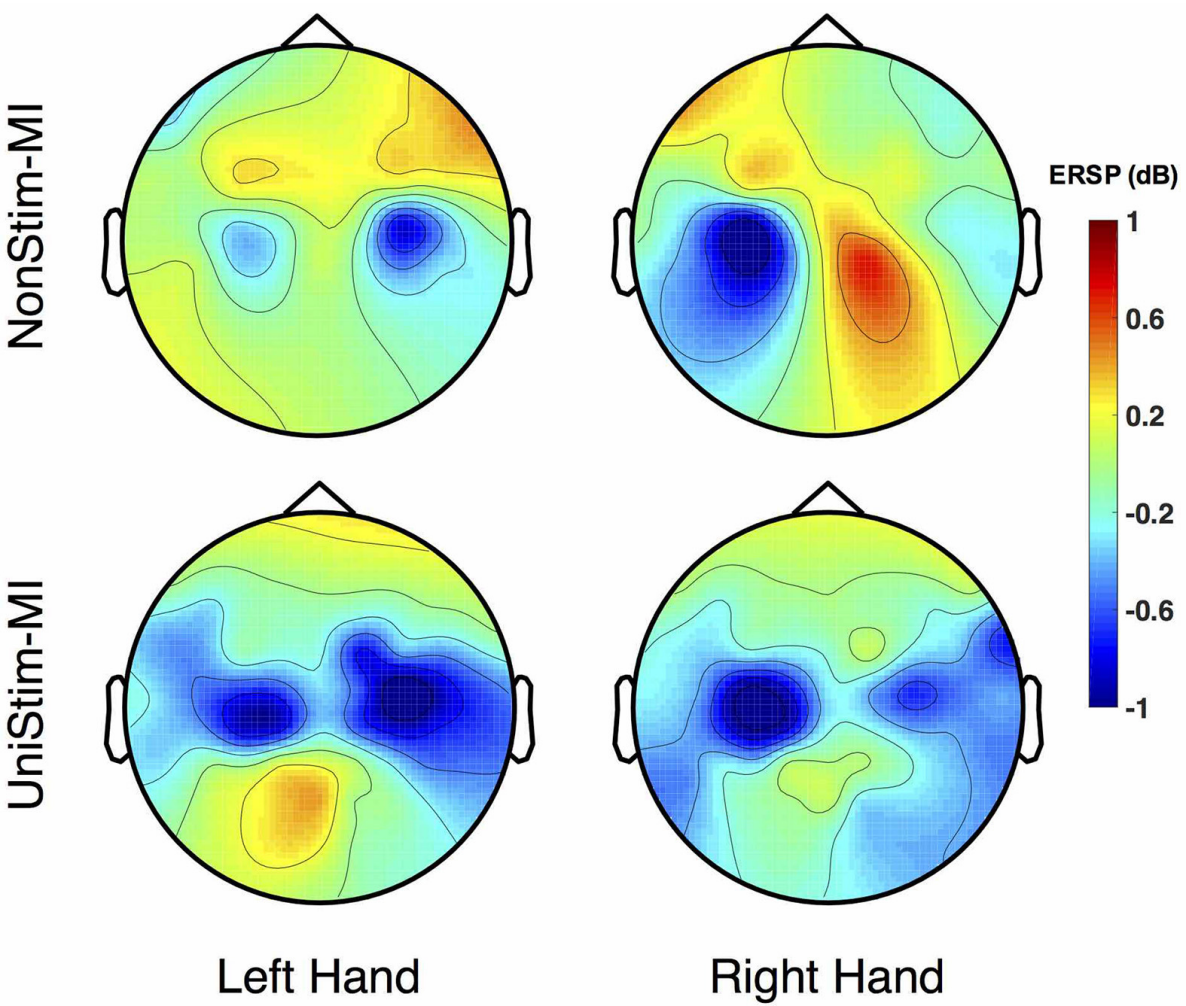
Grand-averaged ERSP distributions of healthy subjects under two different experimental conditions. The ERSP values were calculated within the frequency of [8 30] Hz and timing interval of [1 4] s post-task cues. During NonStim-MI tasks, the contralateral sensorimotor cortices were activated during both left- and right-hand MI, but the contralateral activations were larger for right-hand MI than left-hand MI. However, when UniStim-MI tasks were performed, cortical activations derived from left-hand MI were greatly enhanced, especially in the contralateral hemisphere. Furthermore, the activations corresponding to right-hand MI were not obviously changed, indicating that dominant (right) hand MI was not affected by the stimulation applied on the non-dominant (left) hand. Note that the blue color represents a power decrease and the red color represents a power increase.

The grand-averaged topographic maps for healthy subjects in the enhanced-group are shown in Figure 5. The activation patterns for the NonStim-MI condition is shown in the first row, and the second row is for the UniStim-MI condition. In NonStim-MI tasks, the contralateral sensorimotor cortices were activated during both left- and right-hand MI, but the activations were larger for right-hand MI than left-hand MI. When Uni-TS was applied on the left non-dominant hand, cortical activations in both hemispheres were enhanced during the left-hand MI. However, the cortical activations during right-hand MI were not influenced by the Uni-TS.

**Figure 6:**
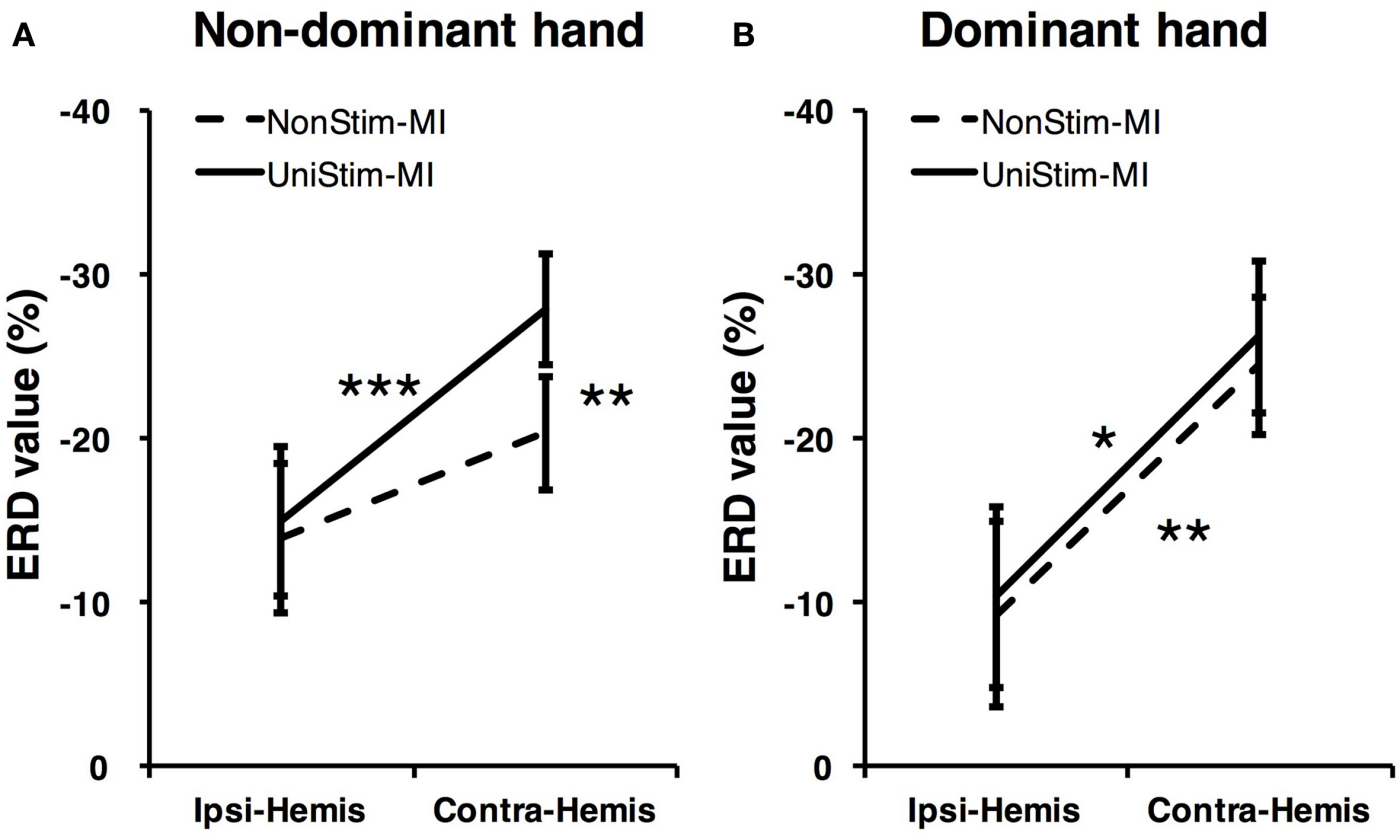
Comparison of grand-averaged ERD values between two hemispheres (Contra-Hemis vs. Ipsi-Hemis) of healthy subjects. **(A)** Comparison of ERD values between contra- and ipsilateral hemispheres during non-dominant hand MI. **(B)** Comparison of ERD values between contra- and ipsilateral hemispheres during dominant hand MI. ^***^p < 0.001, ^**^p < 0.01, and ^*^p < 0.05.

To better understand the effects of Uni-TS on cortical oscillations, the grand-averaged ERD values were compared between the contralateral and ipsilateral hemispheres in Figure 6. During the non-dominant hand MI, the contralateral ERD values were significantly (*p* < 0.001, paired *t*-test) increased when Uni-TS was applied, whereas no significant difference was found for the ipsilateral ERD values between NonStim-MI and UniStim-MI tasks. Moreover, the contralateral ERD values were significantly larger than the ipsilateral ERD values in UniStim-MI (*p* < 0.001, unpaired *t*-test), but no significant inter-hemispheric difference was observed during NonStim-MI tasks. In addition, during the dominant hand MI, neither the ipsilateral nor contralateral ERD values were significantly changed when the Uni-TS was applied. The contralateral ERD values were significantly larger than the ipsilateral ERD values during both NonStim-MI (*p* < 0.01, unpaired *t*-test) and UniStim-MI (*p* < 0.05, unpaired *t*-test) tasks.

### 3.2. Effects of Uni-TS on BCI performance

**Figure 7:**
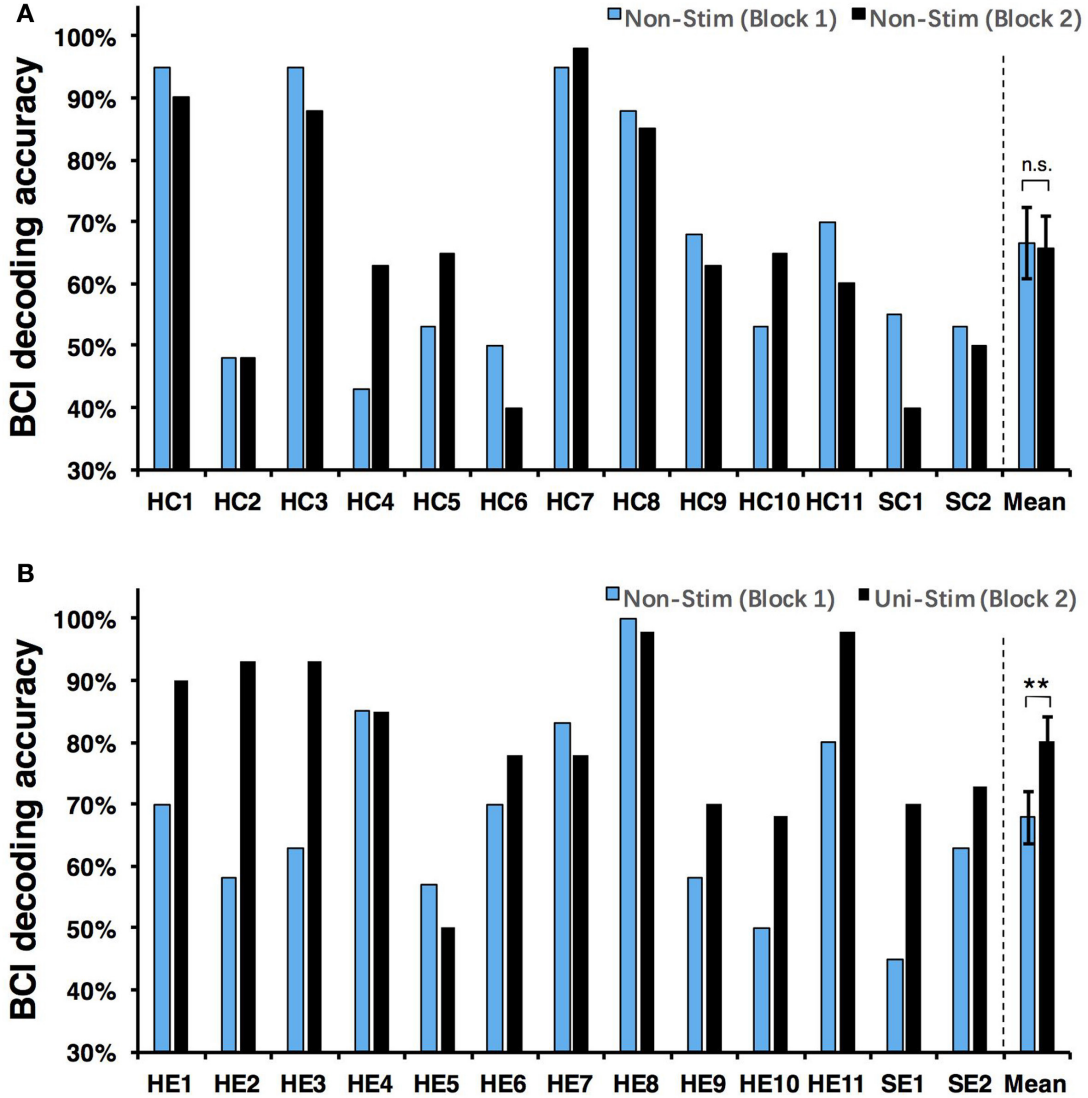
**(A)** On-line decoding accuracy between left- and right-hand MI for the control-group. **(B)** On-line decoding accuracy between left- and right-hand MI for the enhanced-group. The blue and black bars represent decoding accuracies in the first and second blocks. Error bars represent standard error of mean. BCI classifications are performed with EEG signals at [1 4] s post-starting cues, and within frequency band of [8 30] Hz. ^**^p < 0.01 using paired *t*-test, n.s., no significance.

**Figure 8:**
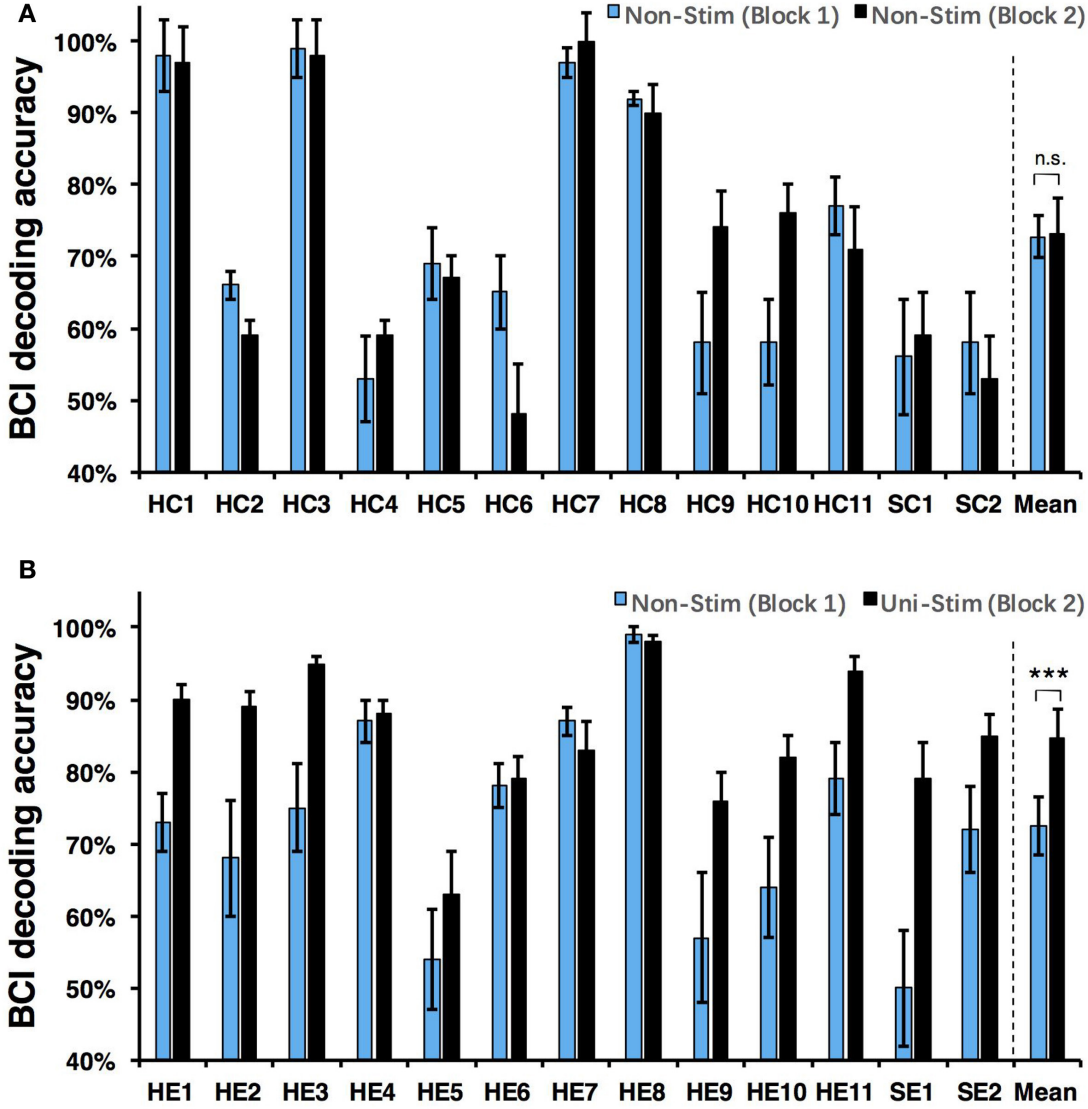
**(A)** Off-line decoding accuracy between left- and right-hand MI for the control-group. **(B)** Off-line decoding accuracy between left- and right-hand MI for the enhanced-group. The blue and black bars represent decoding accuracies in the first and second blocks. Error bars for each individual represent standard deviation, and error bars for mean values represent standard error of mean. BCI classifications are performed with EEG signals at [1 4] s post-starting cues, and within frequency band of [8 30] Hz. ^***^p < 0.001 using paired *t*-test, n.s., no significance.

Both on-line and off-line decoding accuracies were used to assess BCI performance. On-line decoding accuracies of both control-group and enhanced-group are shown in Figure 7. When the Uni-TS was applied, the number of BCI-illiterate users (accuracy < 70%) (Blankertz et al., 2010) decreased from 9 to 2 out of 13 subjects in the enhanced-group, and both stroke patients (SE1, SE2) achieved accuracies above 70%. Moreover, 5 subjects (subject HE1, HE2, HE3, HE8, and HE11) from the enhanced-group achieved accuracies higher than 90% in the UniStim-MI block. However, no significant difference was found between two blocks in the control-group, and 10 subjects were BCI-illiterate users in each block. Off-line analysis results with 10 × 10-fold cross-validation are exhibited in Figure 8. In the enhanced-group, all subjects except subject HE5 achieved an accuracy above 70% in the UniStim-MI block. Specifically, 8 subjects (HE1, HE2, HE3, HE9, HE10, HE11, SE1, and SE2) received an improvement of BCI accuracy ≥ 10%.

We performed a two-way Mixed ANOVA on the on-line and off-line BCI accuracies separately. The dependent variable was BCI accuracy, with time (first block vs. second block) as the within-subjects factor and group (control-group vs. enhanced-group) as the between-subjects factor. The statistical analysis showed a significant time × group [off-line: *F*_(1, 24)_ = 9.516, *p* = 0.005; on-line: *F*_(1, 24)_ = 6.2, *p* = 0.02] interaction and a significant effect of time [off-line: *F*_(1, 24)_ = 10.8, *p* = 0.003; on-line: *F*_(1, 24)_ = 8.138, *p* = 0.009] on BCI accuracies. There was no main effect of group [off-line: *F*_(1, 24)_ = 1.005, *p* = 0.326; on-line: *F*_(1, 24)_ = 1.513, *p* = 0.231]. *Post-hoc* analysis using paired *t*-test was performed to compare the BCI accuracies in different blocks. For the enhanced-group, we found a significant improvement between two MI blocks for both off-line (72.5 vs. 84.7%, *p* < 0.001) and on-line (67.9 vs. 80.3%, *p* < 0.01) BCI accuracies. In contrast, there was no significant difference between two MI blocks in the control-group (on-line: *p* = 0.76; off-line: *p* = 0.88).

**Figure 9:**
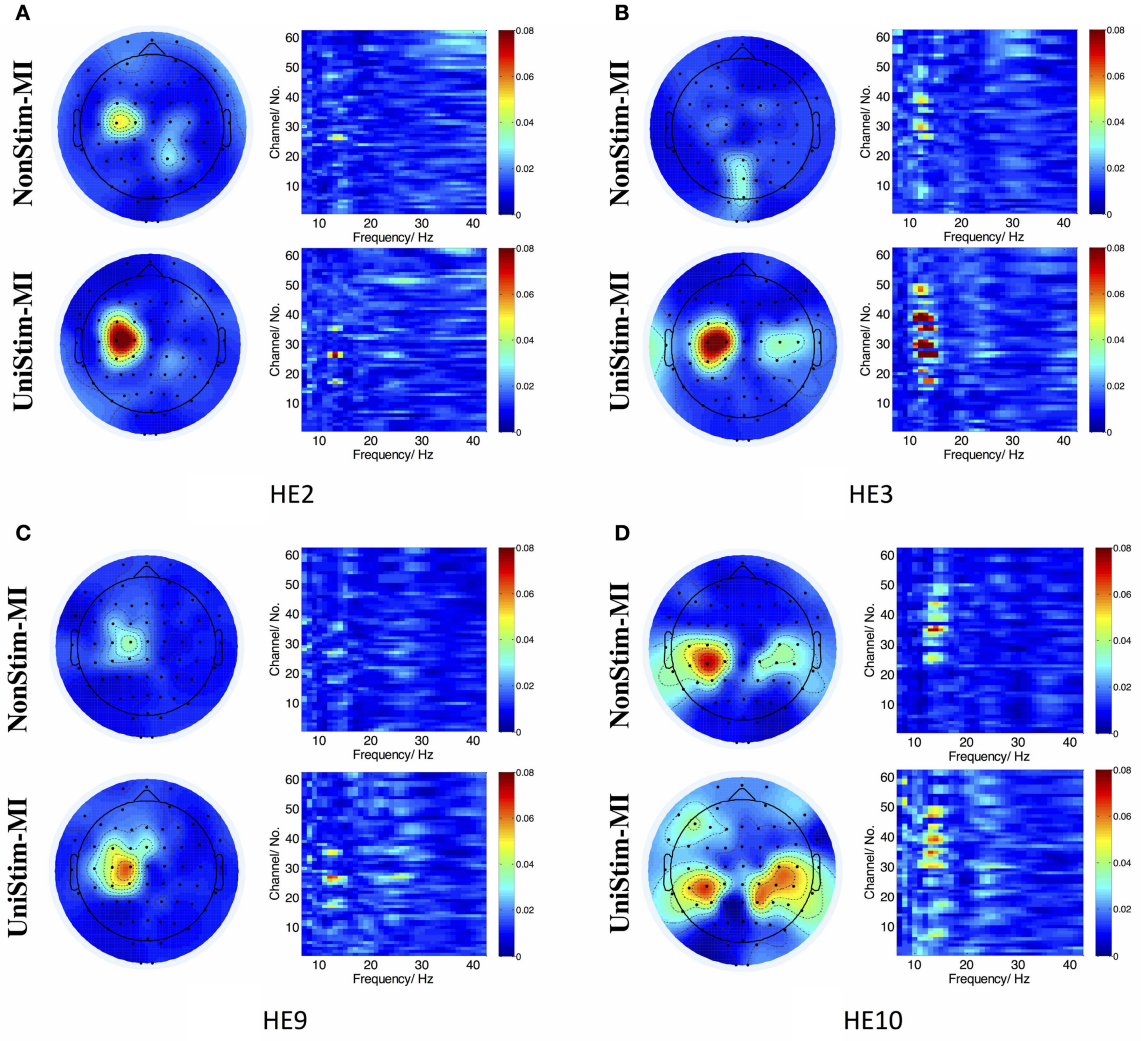
**(A–D)** *R*^2^ value distributions in spatial and frequency domains from healthy subject HE2, HE3, HE9, and HE10, respectively. In each subplot, the upper left and upper right are *R*^2^ value distributions in spatial and frequency domains for NonStim-MI, with the lower left and lower right representing *R*^2^ value distributions in spatial and frequency domains for UniStim-MI. The results elucidated the improvement of BCI decoding accuracy was derived from activations in sensorimotor cortex.

In addition, *R*^2^ coefficients were compared between different conditions. The results of four representative subjects (S2, S3, S9, and S10) are displayed in Figure 9. In each subplot of Figure 9, the upper left and upper right pannels are *R*^2^ value distributions for NonStim-MI, while the lower left and lower right pannels represent *R*^2^ value distributions for UniStim-MI. The results indicated that *R*^2^ coefficients of UniStim-MI were obviously enhanced compared to those of NonStim-MI. Meanwhile, the spatial distribution of *R*^2^-values showed the enhancement was mainly located on the sensorimotor cortices (around channel C3 and C4).

## 4. Discussion

### 4.1. Improvement of MI-BCI performance

The currently available MI-BCIs may have reached the limitation of their performance, as previous research has proved that approximately 30% of healthy subjects (Blankertz et al., 2010) and 40% of stroke patients (Ang and Guan, 2015) can not reach the critical BCI accuracy of 70%. This phenomenon is called “BCI-illiteracy” problem, which has become a big challenge confronting BCI research (Vidaurre and Blankertz, 2010). In the current study, we proposed a novel method for enhancement of MI-BCI by integrating MI with Uni-TS. The Uni-TS was constantly applied on the non-dominant/paretic hand during MI tasks. Inter- and intra-group comparisons of BCI accuracies demonstrated that Uni-TS had beneficial effects on the classification between left- and right-hand MI. As revealed in Figure 8B, most subjects achieved better performance in the UniStim-MI block, except for subject HE7 and HE8 whose decoding accuracies were initially above 80%. The averaged off-line accuracy was significantly increased from 72.5% (NonStim-MI) to 84.7% (UniStim-MI) in the enhanced-group. On the other hand, the proposed approach has partially solved the BCI-illiteracy problem. The number of BCI-illiterate users was decreased from 9 to 2 out of 13 subjects in on-line testing (see Figure 7B). While developing advanced algorithms to extract discriminative features (Ang et al., 2008), training users to modulate cortical activities (Doud et al., 2011) and combining different sensors to form a hybrid BCI (Fazli et al., 2012) have all improved BCI performance, the proposed approach in this study complements the existed methods for BCI enhancement, without increasing source information or complexity of signal processing.

Additionally, the proposed paradigm is expected to benefit stroke patients with damaged motor function but intact somatosensory system. The Uni-TS on paretic hand may improve the MI ability of corresponding hand. Results of two recruited stroke patients (SE1 and SE2) demonstrated the BCI enhancement derived from the Uni-TS. Both patients achieved an accuracy above 80% in UniStim-MI (see Figure 8B). This improvement may facilitate the application of MI-BCI in stroke rehabilitation. Bundy et al. (2017) demonstrated that higher BCI accuracies induced better rehabilitation performance. Thus, the Uni-TS aided BCI system is expected to outperform the conventional BCI systems in stroke rehabilitation.

Tactile stimulation for BCI enhancement has been investigated in previous works. Yao et al. (2014) combined motor imagery with tactile sensation to form a hybrid BCI system. Subjects were instructed to perform either MI or tactile sensation when vibrotactile stimulations were simultaneously applied on both hands. Interestingly, the two-class BCI accuracy was significantly improved from 71.9% in the conventional MI-BCI to 83.1% in the hybrid modality (right-hand MI & left-hand sensation). This strategy coincided with the current paradigm when MI was integrated with Uni-TS specifically on the left hand. However, the mechanism for enhancement in this study was greatly different from the hybrid approach. Physiologically, MI with Uni-TS in this work should have activated both motor and sensory cortices, but tactile sensation in Yao et al. (2014) only activated the sensory cortex and involved different neural pathways in these two methods (Dockstader et al., 2010). In another study, Chatterjee et al. (2007) applied vibrotactile stimulation on a single hand as continuous feedback for BCI-control, and BCI accuracy was utilized to evaluate the system performance. The results indicated that the BCI accuracy was improved when the stimulation was applied on the ipsilateral hand. Furthermore, improvement of the left-hand MI was much higher than that of the right-hand MI (70 vs. 58%) when stimulations were applied on the imagined hand. It demonstrates that the non-dominant hand MI benefits more from the somatosensory stimulation. Gandrey et al. (2013) proved that dominant hand was superior to non-dominant hand in MI tasks. Therefore, the Uni-TS on non-dominant hand may balance the MI abilities between dominant and non-dominant hand, and consequently improve the BCI accuracy between different mental tasks.

### 4.2. Electrophysiological evidence for BCI enhancement

Cortical activities during hand MI are generally lateralized to the contralateral hemisphere in healthy subjects (Doyle et al., 2005; Nam et al., 2011; Zich et al., 2015). The grand-averaged topographic maps in Figure 5 are partially consistent with this phenomenon, i.e., during the left-hand MI the ERSP in the right hemisphere is larger as compared to the ERSP in the left hemisphere, and vice versa for the right-hand MI. However, statistical analysis of ERD values presented significant inter-hemispheric difference only for the dominant hand MI, but not for the non-dominant hand MI (see Figure 6). Moreover, spectral analysis of a representative subject (S3) showed that MI-induced cortical activities were lateralized to the ipsilateral side for the non-dominant hand (see Figure 3A). This finding is in consistence with (Bai et al., 2005) that activations in contralateral hemisphere were greater during dominant hand tasks compared with non-dominant hand tasks. Other studies also reported significant ipsilateral activations during non-dominant hand tasks (Kawashima et al., 1993, 1994; Porro et al., 2000; Fujiwara et al., 2017). This observation could be explained by the inhibitory theory, i.e., inter-hemispheric inhibition from contralateral to ipsilateral motor cortices is stronger for dominant hand tasks than non-dominant hand tasks (Netz et al., 1995). Therefore, the lateralization of cortical activations during non-dominant hand tasks was reduced, which may decrease the BCI decoding accuracy.

As expected, Uni-TS on the non-dominant hand enhanced the cortical activations in the contralateral hemisphere as shown in Figure 5. It implies that an appropriate sensory afferent stimulation could improve the vividness of MI. Actually, the interaction between motor imagery and sensory afferent occurs in the contralateral motor cortex (M1) and sensory cortex (S1) (Gandolla et al., 2014). This interaction enhances the activations in S1 and M1 areas. For instance, the cortical excitability during MI could be enhanced by voluntarily touching and feeling a real object with the imagined hand (Mizuguchi et al., 2009). Meanwhile, due to the lateral inhibition (Severens et al., 2010), the cortical excitability induced by sensory afferent was suppressed during the opposite hand MI. Therefore, the Uni-TS applied on non-dominant hand balanced the cortical activations between left- and right-hand MI. Grand-averaged ERD values in Figure 6 further confirmed that the contralateral activities during non-dominant hand MI were significantly enhanced when the Uni-TS was applied. Conversely, the activations during dominant hand MI were not significantly changed. In this way, ERD lateralization of hand MI was increased. These changes of cortical activations via Uni-TS consequently improved MI-BCI accuracies, because stronger ERD lateralization was associated with higher single-trial decoding accuracies (Nikulin et al., 2008).

### 4.3. Clinical implication and future works

Although BCI decoding accuracy was mainly covered in this study, from the aspect of stroke rehabilitation, enhancement of cortical activations may be more important. As reported in previous work, MI-induced cortical activations in stroke patients were significantly decreased when compared to age-matched healthy adults, especially for the lesioned hemisphere (Park et al., 2015). Thus, enhanced cortical activations in the lesioned motor cortex may serve as an indication of recovery from motor deficit (Johansen-Berg et al., 2002; Calautti et al., 2007). As shown in Figure 4, Uni-TS applied on the paretic hand enhanced the contralateral activations during paretic hand MI, which was considered to have benefits on neural reorganization after stroke. Thus, the proposed stimulation strategy may not only enhance the BCI performance, but also have specific benefits on neural repair after stroke. Clinical evidence also revealed that somatosensory stimulation (e.g., vibrotactile stimulation, or electrical stimulation) could improve the training effects of motor function in chronic stroke patients (Sawaki et al., 2006; Conforto et al., 2007; Bastos Conforto et al., 2010). These results implied that the Uni-TS aided MI-BCI was more suitable for stroke rehabilitation than the conventional MI-BCI paradigm.

However, due to the small sample size of stroke group, the effects of Uni-TS on cortical activations are still largely unrevealed in stroke patients. In future works, more patients should be recruited to test the effects of a constant Uni-TS on the cortical activations and BCI performance. Furthermore, we are going to associate the Uni-TS with brain activities to close the sensorimotor loop. This experimental design is expected to be valuable for stroke rehabilitation. Long-term experiments on stroke patients are required to evaluate the clinical effects of this approach.

## 5. Conclusion

In this work, we explored the effects of Uni-TS on MI-BCI. We found the Uni-TS significantly enhanced the contralateral cortical activations with respect to non-dominant/paretic hand MI. These physiological changes led to a great improvement of two-class BCI accuracy. Meanwhile, the recruited stroke patients also exhibited evident improvement in BCI performance when the Uni-TS was applied. This proposed approach showed a great potential to make MI-BCI more applicable to a wider range of BCI users.

## Author contributions

XShu and LY conceived and designed the experiment paradigm. XShu performed the experiments and wrote the manuscript. XShe, DZ, and XZ reviewed and edited the manuscript. All the authors read and approved the manuscript.

### Conflict of interest statement

The authors declare that the research was conducted in the absence of any commercial or financial relationships that could be construed as a potential conflict of interest.
